# G45R mutation in the nonstructural protein 1 of A/Puerto Rico/8/1934 (H1N1) enhances viral replication independent of dsRNA-binding activity and type I interferon biology

**DOI:** 10.1186/s12985-016-0585-4

**Published:** 2016-07-12

**Authors:** Challika Kaewborisuth, Mark Zanin, Hans Häcker, Richard J. Webby, Porntippa Lekcharoensuk

**Affiliations:** Interdisciplinary Graduate Program in Genetic Engineering, The Graduate School, Kasetsart University, Bangkok, 10900 Thailand; Department of Infectious Diseases, Division of Virology, St. Jude Children’s Research Hospital, Memphis, 38105-2794 TN USA; Department of Infectious Diseases, St. Jude Children’s Research Hospital, Memphis, 38105-2794 TN USA; Department of Microbiology and Immunology, Faculty of Veterinary Medicine, Kasetsart University, 50th Ngamwongwan Rd., Chatuchak, Bangkok, 10900 Thailand; Center for Advances Studies in Agriculture and Food, KU Institute for Advanced Studies, Kasetsart University, Bangkok, 10900 Thailand

**Keywords:** Influenza A virus, NS1, G45R, dsRNA binding, Type I IFN, Virus replication

## Abstract

**Background:**

The nonstructural protein 1 (NS1) of influenza A viruses can act as a viral replication enhancer by antagonizing type I interferon (IFN) induction and response in infected cells. We previously reported that A/Puerto Rico/8/1934 (H1N1) (PR8) containing the NS1 gene derived from A/swine/IA/15/1930 (H1N1) (IA30) replicated more efficiently than the wild type virus. Here, we identified amino acids in NS1 critical for enhancing viral replication.

**Methods:**

To identify a key amino acid in NS1 which can increase the virus replication, growth kinetics of PR8 viruses encoding single mutation in NS1 were compared in A549 cells. NS1 mutant functions were studied using dsRNA-protein pull down, RIG-I mediated IFNβ-promoter activity assays and growth curve analysis in murine lung epithelial type I (Let1) cells.

**Results:**

The G45R mutation in the NS1 of PR8 (G45R/NS1) virus is critical for the enhanced viral replication in A549 cells. G45R/NS1 slightly decreased NS1 binding to dsRNA but did not interfere with its suppression of RIG-I-mediated type I IFN production. Likewise, replication of G45R/NS1 virus was increased in comparison to wild type virus in both wild type and type I interferon receptor null Let1 cells.

**Conclusions:**

The non-conserved amino acid, R45, enhances viral replication which is apparently independent of dsRNA binding and suppression of type I IFN, suggesting a non-characterized function of NS1 for the enhanced viral replication. As G45R/NS1 virus induced the type I IFN induction and response in infected A549 cells, it is also interesting to investigate virus virulence for further studies.

**Electronic supplementary material:**

The online version of this article (doi:10.1186/s12985-016-0585-4) contains supplementary material, which is available to authorized users.

## Background

Influenza A virus has evolved a variety of mechanisms to evade host cell defenses to facilitate efficient transmission [1, 2], replication and virulence [3]. Escape from host innate immune responses allows influenza A virus to proficiently replicate in the cells. The most important innate immune response against influenza A virus is the interferon (IFN) system [4]. There are three types of IFN families including type I (−α, − β, −ω and -ɛ), type II (−γ) and type III (−λs or IL28/29) [5]. Type I IFNs, especially -α/β, are essential for host defense against viral infection. It is induced following the recognition of viral components or RNA by pathogen recognition receptors (PRRs) such as retinoic acid-inducible gene I (RIG-I) in the cytoplasm and toll-like receptors (TLR) in the endosome and on the cell surface [6–8].

IFN signaling is mediated via binding between IFN and its receptor, which triggers downstream signaling via Janus Activated Kinase (JAK) and Tyrosine Kinae-2 (TYK2) followed by Signal Transducer and Activators of Transcription (STAT) proteins [9]. After activation, STATs translocate into the nucleus to initiate transcription of IFN-stimulated genes (ISGs), such as 2’-5’oligoadenylate synthetase (OAS1), dsRNA-dependent protein kinase R (PKR) and Mx1, which have antiviral functions [10, 11].

The non-structural protein 1 (NS1) of influenza A virus plays an important role in counteracting the IFNα/β system [12], thereby preventing the generation of an antiviral state in the cell and facilitating efficient viral replication [13, 14]. This action of NS1 was shown at multiple levels in vitro and is different among influenza A virus strains [15]. NS1 contains two major functional domains, an N-terminal RNA-binding domain (RBD), which binds dsRNA, and a C-terminal effector domain (ED). The RBD has been proposed to sequester dsRNA from RIG-I [16] and dsRNA-activated antiviral enzymes such as OAS1 [17] and PKR [18, 19] to prevent their antiviral activities.

Previously, we reported that reassortant viruses containing internal gene segments derived from the A/swine/IA/15/30 H1N1 virus (IA30) with the hemagglutinin (HA) and neuraminidase (NA) from the avian influenza virus H5N1 or swine influenza virus H3N2 replicated more efficiently than those with internal gene segments from A/Puerto Pico/8/1934 (H1N1) (PR8) in Madin Darby canine kidney (MDCK) and Vero cells [20]. Among the IA30 genes, IA30 NS has been shown to increase PR8 virus replication more efficiently than wild type PR8 NS1 [21]. In this report, we have identified a non-conserved residue of NS1 that enhanced viral replication independent of its dsRNA-binding ability and the IFN antagonist function.

## Results

### The RNA-binding domain of IA30 NS1 increased virus replication

**Fig. 1:**
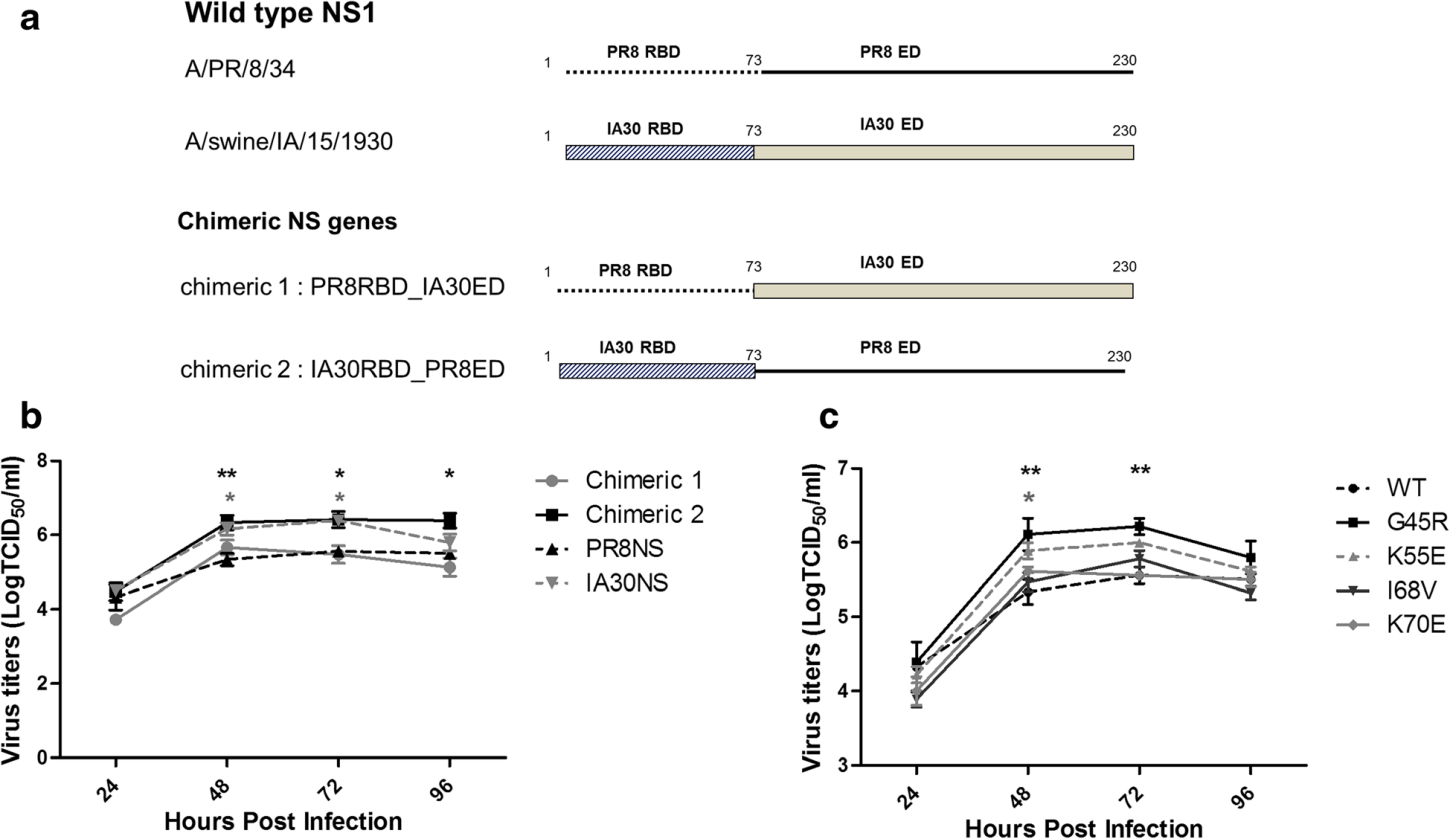
Schematic diagram of wild type and chimeric NS1 proteins and growth kinetics of reverse genetics viruses, A/Puerto Rico/8/1934 (PR8) with X31 HA and NA (rX31) encoding wild type (WT) and mutated nonstructural protein 1 (NS1). **a** Wild type and chimeric NS1 proteins (230 amino acids in length) contain the RNA binding domain (RBD) (amino acid 1–73) and effector domain (EF) (amino acid 74–230). Chimera 1 contains the RBD derived from PR8 NS1 and the ED derived from A/swine/IA/15/1930 NS1 (IA30 NS1) whilst Chimera 2 contains the RBD derived from IA30 NS1 and the ED derived from PR8. **b** The viruses containing PR8 NS1, IA30 NS1, chimera 1 or chimera 2, and **c** PR8 NS1 with a single amino acid mutation (G45R, K55E, I68V and K70E) were infected in triplicate in A549 cells at an MOI of 0.01. Culture medium was collected every 24 h post infection for virus titration by TCID_50_ /ml in Madin-Darby canine kidney (MDCK) cells. *, ** and *** represent the statistically significant differences of mean titers compared to the WT at *p* < 0.05, *p* < 0.01 and *p* < 0.001, respectively, as determined by ANOVA

We reported previously that IA30 replicated to higher titers than PR8 [20] and it was mediated by the IA30 NS [21]. In this study, we compared the growth kinetics of viruses generated by reverse genetics based on X31 containing the HA and NA of A/Hong Kong/1/1968 (H3N2), the PB2, PB1, PA, NP, and M of PR8 and the NS of either PR8 or IA30. The virus containing IA30 NS grew to significantly higher titers at 48 and 72 h post infection (hpi) than the virus containing the PR8 NS (Fig. 1b). To identify the IA30 NS1 domain responsible for increasing viral replication, we generated viruses containing chimeric NS genes. Chimera 1 contained the RBD derived from PR8 NS1 and the ED derived from IA30 NS1 whilst Chimera 2 contained the RBD derived from IA30 NS1 and the ED derived from PR8 (Fig. 1a). Chimera 2 reached significantly higher titers compared to PR8 NS1 virus, whilst Chimera 1 had a comparable replication rate to the PR8 NS1 virus (Fig. 1b). These results indicated that the IA30 RBD was involved in increasing viral replication.

We performed multiple alignments of the deduced amino acids of both chimeras. The result shows that few amino acids on NS2 of both chimeras are different. NS2 has a nuclear export signal and interacts with M1 via its C-terminal domain (aa 81–100) to transport the vRNP from nucleus to cytoplasm for viral assembly (Shimizu et al., 2011) and also regulates the accumulation of viral RNA species including viral RNA, cRNA and mRNA, which are important for viral transcription and replication (Robb et al., 2009). According to NS2 functions, we cannot exclude that NS2 translated from chimeric 1 and 2 genes may have an effect on the replication.

### A single amino acid substitution, G45R, in PR8 NS1 contributed to enhanced viral replication

To determine amino acids in the RBD of IA30 NS1 that contributed to increased viral replication, amino acid sequences of PR8 and IA30 NS1s were compared. Eight residues in the RBD were different between the two strains. We selected four point mutations at positions 45, 55, 68 and 70, which did not affect NS2 expression, to introduce into the RBD coding region of PR8 NS1 to generate viruses containing PR8 NS1 with G45R, K55E, I68V or K70E mutations via reverse genetics [22]. Growth kinetics of the viruses was assessed in A549 cells at a multiplicity of infection (MOI) of 0.01. Viruses with NS1 mutations at G45R and K55E (G45R and K55E/NS1s) grew to higher titers compared to the wild type (WT), I68V and K70E/NS1s viruses. The G45R/NS1 virus replicated to the highest titer (*P* ≤ 0.001) at 48 to 96 hpi while the replication rates of I68V and K70E/NS1s mutants were similar to the WT/NS1 virus (Fig. 1b). The increased virus replication with K55E substitution in PR8 NS1 has been addressed previously for cell-based vaccine production [23]. As the G45R/NS1 virus replicated to the highest titer, it was selected for further studies.

### G45R/NS1 did not increase binding to dsRNA in vitro

**Fig. 2:**
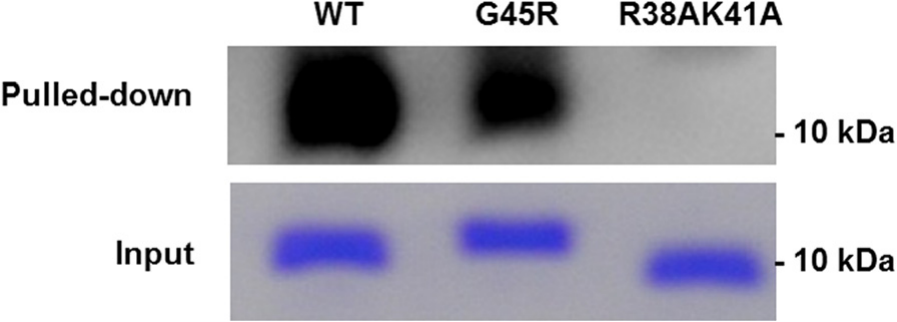
dsRNA-RBD NS1 pull down assay. The G45R/NS1 bound to dsRNA with lower affinity than the wild type (WT). The R38AK41A as a negative control failed to bind with dsRNA. The input NS1_RBDs for dsRNA-protein pulled down assay were loaded and run onto 15 % acrylamide gel. The input proteins were detected by Coomassie brilliant blue staining

The RBD of NS1 binds to dsRNA to counteract the host IFN system and facilitate virus replication [16, 17]. As viruses containing G45R/NS1 replicated at the highest rate, we sought to elucidate its impact on RNA binding activity in vitro. To assess the dsRNA binding ability of the G45R/NS1 protein, the RBD of PR8 NS1 [WT, G45R and the dsRNA binding deficient mutant R38AK41A (AA)] [13] were expressed in *E. coli*. The expressed proteins were purified by affinity chromatography and used in the dsRNA-protein pull down assay. WT/NS1 bound to dsRNA with high affinity while the deficient mutant, AA/NS1, failed to bind dsRNA (Fig. 2). However, G45R/NS1 did not increase the binding ability of NS1 compared to WT/NS1 (Fig. 2). All three replicates of the pull down reactions performed separately yielded similar results (Additional file 1: Fig. S1). Therefore, G45R/NS1 does not play a significant role in the dsRNA-NS1 interaction in vitro.

### G45R/NS1 did not increase inhibition of RIG-I mediated IFNβ-promoter activation

**Fig. 3:**
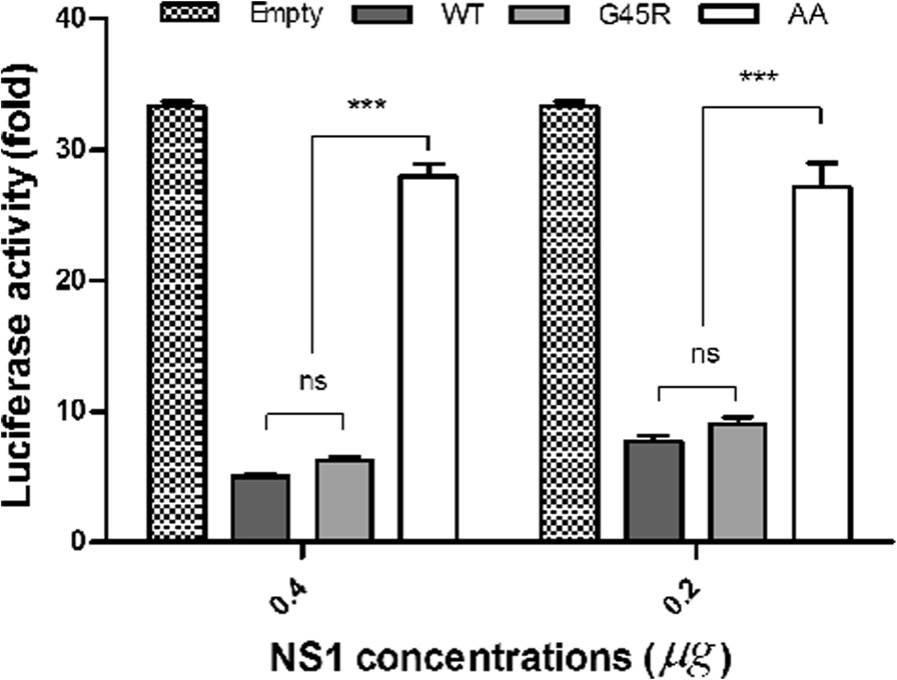
RIG-I mediated IFNβ-promoter activity in 293 T cells expressing PR8 NS1 (wild type; WT, G45R and R38AK41A; AA), RIG I and IFNβ-promoter luciferase reporter at 24 h post infection. WT and G45R NS1s decreased the luciferase expression in dose-dependent manner by inhibition of RIG-I mediated IFNβ-promoter activity. The double mutant R38AK41A served as a negative control failed to inhibit RIG I mediated luciferase expression via IFNβ-promoter. *** represents the statistically significant difference of mean luciferase activity compared to the WT and G45R at *p* < 0.001, as determined by ANOVA

RIG-I is a cytoplasmic pathogen sensor that is crucial to recognize viral RNA, particularly 5’-ppp ssRNA and dsRNA, and trigger type I IFN induction to combat viral infection [24, 25]. NS1 has been shown to inhibit RIG-I-mediated IFN induction by forming a complex with RIG-I partly via the RNA-binding domain [26]. We assessed whether G45R/NS1 was able to inhibit RIG-I mediated IFN synthesis in 293 T cells co-transfected with plasmids including pCAGGS_PR8NS1 (WT, G45R or AA) or empty pCAGGS as a control, pRIG-I and pfirefly luciferase driven by the IFNβ promoter and p*Renilla* Luciferase. RIG-I mediated IFNβ-promoter activation was measured by dual luciferase reporter assay. The IFNβ promoter was strongly activated in cells transfected with RIG-I; however, in the presence of NS1, IFNβ activation was reduced. AA/NS1 was less efficient in suppressing RIG-I-mediated IFNβ-promoter activity, as expected (Fig. 3). The inhibition of RIG-I mediated IFNβ-promoter activity by WT and G45R/NS1 proteins were similar while the NS1 expressions by each virus in the transfected cells were not different (Additional file 2: Fig. S2). This confirmed that the different luciferase signals did not come from different levels of NS1 expression. Therefore, the increased replication of G45R/NS1 virus was not due to NS1-mediated alterations of RIG-I mediated IFNβ-promoter activation.

### G45R/NS1 increased virus replication independent of type I IFN induction

**Fig. 4:**
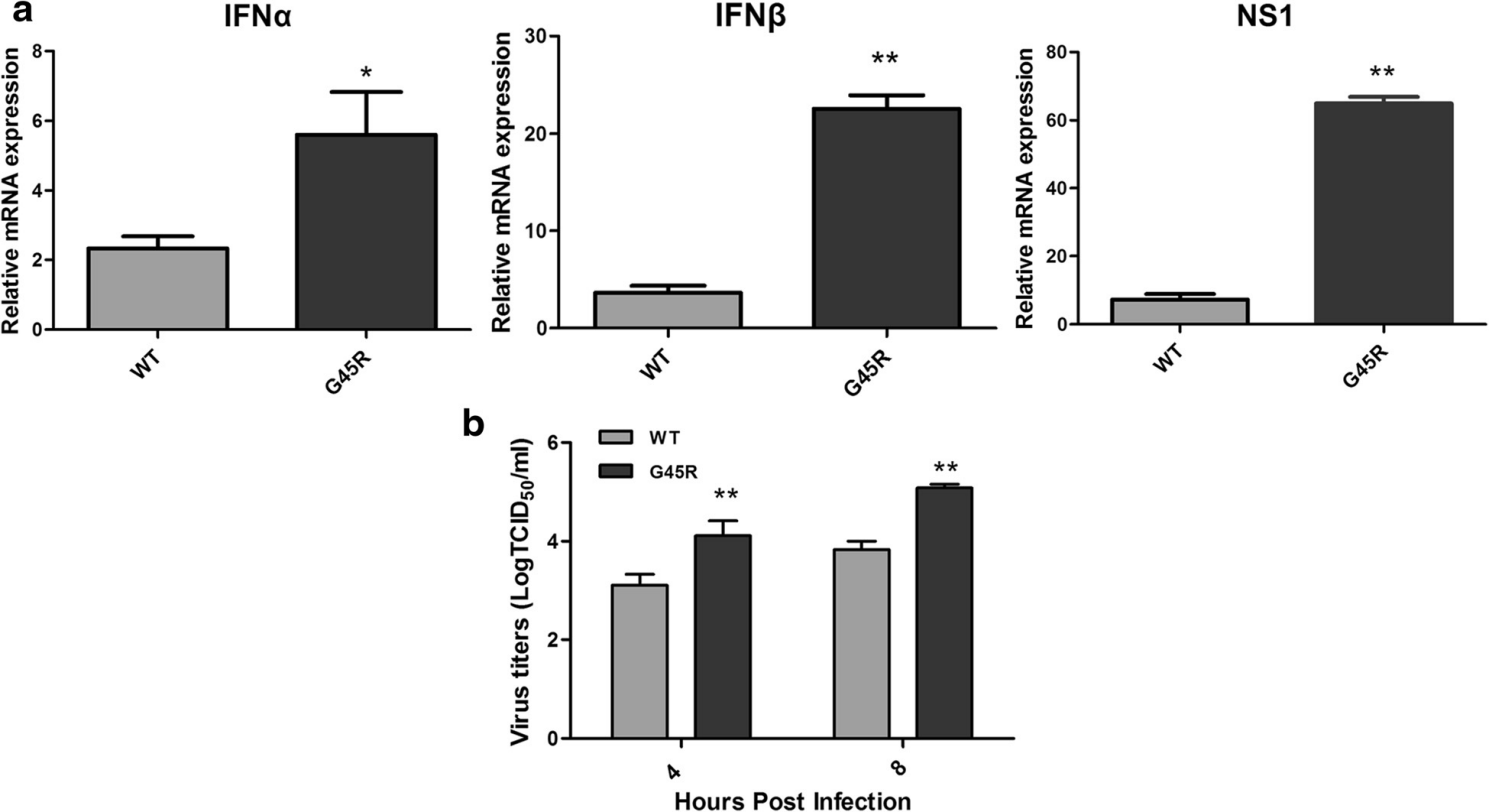
Expression of type I IFN and NS1 in rX31 infected A549 cells and rX31 growth kinetics. **a** The expression levels of IFNα, IFNβ and NS1 in A549 cells infected with rX31 virus encoding WT or G45R NS1 at 8 h post infection. rX31 with G45R NS1 induced significantly high level of IFNα, IFNβ and NS1 expression compared to the WT virus. Graphs represent mean ± SEM, * and ** represent the statistically significant difference of mean relative mRNA expression compared to the WT at *p* < 0.05 and *p* < 0.01, respectively, as determined by student *t*-test. **b** Growth of rX31 viruses encoding PR8 NS1 (WT and G45R). rX31 virus with G45R NS1 also replicated to significantly high titer compared to the WT virus early in infection. ** represents the statistically significant difference of mean titers compared to the WT at *p* < 0.01, as determined by ANOVA

Our data suggested that the anti-RIG-I activity of the G45R/NS1 variant was similar to WT NS1. To test this hypothesis, we infected A549 cells with WT and G45R/NS1 viruses at an MOI of 1 to measure type I IFN mRNA expression and single step growth kinetics. As shown earlier, the G45R/NS1 virus replicated more efficiently than the WT virus, which is particularly apparent early in infection. Interestingly, the G45R/NS1 virus induced greater expression of IFNα/β mRNA compared to the WT/NS1 (Fig. 4). As such, G45R/NS1 did not suppress type I IFN expression to facilitate virus replication.

**Fig. 5:**
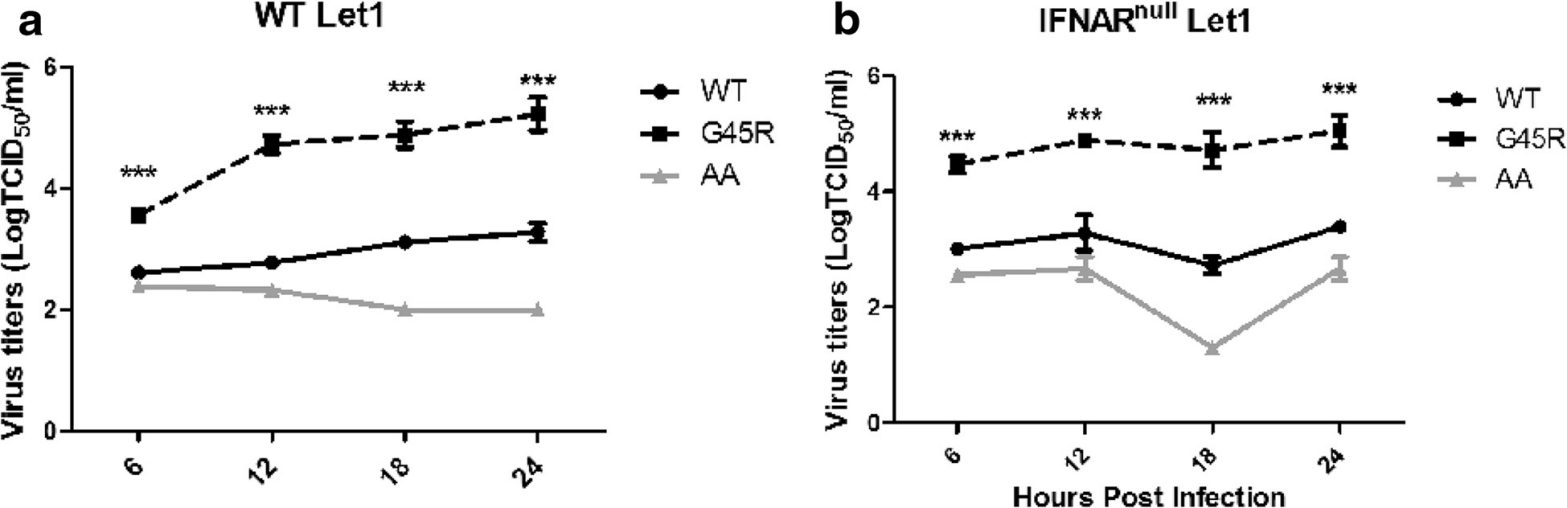
Replication kinetics of the rX31 virus encoding PR8 NS1s (WT, G45R or R38AK41A (AA)) in wild type (WT) and type I IFN receptor null (IFNAR^null^) Let1 cells. rX31 with G45R replicated to significantly higher titer that WT in both **a** WT and **b** IFNAR^null^ Let1 cells. The virus with RNA binding deficient NS1, AA, could not replicate well in both cell types. *** represents the statistically significant difference of mean titers compared to the WT at *p* < 0.001, as determined by ANOVA

Based on these observations we concluded that G45R/NS1 does not possess type I IFN suppressive activity and hence is not responsible for the increased G45R/NS1 virus replication. To further evaluate the relevance of type I IFNs for virus replication, we conducted growth kinetics experiments using WT, G45R and AA/NS1s viruses in WT and type I interferon receptor null (IFNAR^null^) murine lung epithelial type I (Let1) cells [27]. If type I IFNs were involved in the difference between the replication kinetics of WT and G45R/NS1 viruses, replication of the G45R/NS1 virus should be greater than the WT/NS1 virus in WT Let1 cells yet similar in IFNAR^null^ Let1 cells. As shown in Fig. 5, the G45R/NS1 virus replicated to higher titers compared to the WT/NS1 virus in both WT and IFNAR^null^ Let1 cells at every time point investigated, further supporting our conclusion that modulation of type I interferon biology, at the level of RIG-I-induced IFN activation or at later levels of type I interferon activity, is not the reason for the increased replicative capacity of the G45R/NS1 virus observed.

## Discussion

The NS1 protein of influenza A virus is multifunctional and associated with increased viral pathogenicity and replication by counteracting host antiviral defense mechanisms [28]. NS1 inhibits IFNα/β induction and the antiviral response through different pathways [4, 15, 29–31], though some functions are strain-specific [32, 33]. It was shown previously that recombinant viruses with different HA and NA subtypes in an IA30 virus background had much higher replication rates compared to viruses in a PR8 virus background [20]. Recently, IA30 NS has been shown to involve in the increased virus replication [21]. Here, we investigated the roles of amino acids in IA30 NS1 in enhancing viral replication. We constructed viruses using reverse genetics that contained the HA and NA genes from X31 in a PR8 background and demonstrated that the single amino acids substitution, G45R, in the RBD of PR8 NS1 accelerated viral replication.

The RBD of NS1 plays a major role in inhibiting host defense mechanisms, in particular type I IFN production. It may sequester dsRNA from PRRs and ISG products that require activation by dsRNA, such as RIG-I, PKR and OAS1 [4, 17, 30]. In addition, NS1 also interacts with these proteins to block their functions [19, 24]. Viruses containing the R38A mutation in NS1 were attenuated, lacked dsRNA binding and were sensitive to IFNβ [23]. However, replication of viruses containing the R38A NS1 mutation was improved in RNaseL knockout cells, suggesting that NS1 was involved in the inhibition of the 2’-5’ OAS/RNaseL pathway, possibly by sequestering dsRNA and preventing activation of OAS1 [23].

Previously, *in silico* analysis suggested that the R45 on NS1 of the pandemic virus A/Texas/15/2009 (H1N1) increased the stability of the dsRNA-NS1 complex, which contributed to viral pathogenicity [34]. We investigated whether the enhanced viral replication mediated by G45R/NS1 was due to the increased dsRNA binding in vitro using dsRNA-NS1 pull-down assays. In these assays, G45R/NS1 did not show increased dsRNA-binding compared to WT/NS1. As G45R/NS1 did not act as predicted [34], we speculated that substitution of G45R on PR8 NS1 may impact dsRNA binding by steric hindrance. Nonetheless, NS1 functions do not depend solely on its dsRNA-binding activity. WSN-NS1 with the triple mutations R38A, K41A and S42G could not bind to dsRNA similar to R38A and K41A, though the virus containing R38A, K41A and S42G decreased activation of the IFNβ promoter and had a higher replication rate compared to the virus containing R38A and K41A [13].

RIG-I is a crucial cytoplasmic sensor for dsRNA and 5’ppp-ssRNA that triggers downstream signaling to activate type I IFN production during virus infection [24, 25]. It has been reported that NS1 interferes with the RIG-I mediated IFN pathway partly through its RBD, increasing viral replication and pathogenicity [35, 36]. We investigated whether G45R/NS1 increased virus replication by inhibiting RIG-I mediated IFNβ-promoter activation. The reporter assay showed that G45R/NS1 suppressed the activation of IFNβ-promoter comparable to WT/NS1 (Fig. 3). We suggest that the G45R mutation in PR8 NS1 facilitated virus replication independent of dsRNA-binding and RIG-I mediated IFNβ promoter activation.

We confirmed that type I IFNs induction was not relevant to G45R/NS1 virus replication by using IFNAR^null^ Let1 cells. G45R/NS1 replicated to higher titers compared to WT virus while AA/NS1 virus was attenuated in both WT and IFNAR^null^ Let1 cells (Fig. 5) suggesting that G45R/NS1 strongly influenced virus replication in a type I IFN induction-independent manner. In fact, high replication of G45R/NS1 was accompanied by increased type I IFN and STAT1 phosphorylation (Fig. 4 and Additional file 3: Fig. S3), conferring a strong activation of type I IFN signaling. Elevation of type I IFN expression can up-regulate the expression of various cytokines and chemokines to recruit immune cells to the site of infection. In addition, IFNα has been reported to enhance CCL5 and CCL10 expression in influenza virus-infected A549 cells [37]. Rapid virus replication, systemic spread and activation of IFNα/β signaling by influenza virus infection could lead to excessive production of cytokines and chemokines, resulting in increased morbidity and mortality in mice [38, 39]. An adaptation of amino acids on NS1, F103L and M106I, of human influenza A/Hong Kong/1/68(H3N2) has been reported to be associated with the increased virus replication and virulence in mice and human [40] thereby the occurrence of G45R mutation on NS1 of pandemic human 2009 H1N1 virus might be one of crucial factors to cause severe disease. Even though G45R in NS1 presents in a minor population of the pandemic H1N1 2009 viruses, it has been proposed that G45R mutation might be related to the increased cytokine production observed during infections by these viruses [41].

## Conclusions

We elucidated the function of the non-conserved amino acid, R45, in NS1, which facilitated viral replication by an undefined mechanism. G45R/NS1 does not increase the efficiency of binding to dsRNA or suppress IFNβ induction, which are the established functions of NS1. Thus, increased viral replication of G45R/NS is independent of dsRNA binding and type I IFN induction, suggesting that a non-characterized function of NS1 was responsible for the enhanced viral replication observed in this study. As we have demonstrated that G45R/NS1 virus induced the type I IFN induction and response in infected A549 cells, it is also interesting to investigate virus virulence for further studies.

## Methods

### Cells lines

Human embryonic kidney (293 T), human lung carcinoma (A549) and Mardin-Darby canine kidney (MDCK) cells were cultured in Opti-MEM I reduced serum medium (Gibco, Invitrogen), Kaighn’s Modification of Ham’s F-12 Medium (ATCC) or minimum essential media (Invitrogen), respectively, supplemented with 5 % fetal bovine serum (Invitrogen), L-glutamine (Invitrogen), penicillin and streptomycin (Invitrogen). Murine lung epithelial type I cell line (Let1; wild type or type I IFN receptor null, IFNAR^null^), kindly provided by Alan Aderem and Alan Diercks [27], was cultured in Dulbecco’s Modified Eagle Medium (Invitrogen) supplemented with 10 % fetal bovine serum, penicillin and streptomycin. All cells were incubated in a humidified atmosphere of 5 % CO_2_ at 37 °C.

### Construction of plasmids

Two chimeric NS genes—chimera 1 and chimera 2—comprising the 3’UTR and RBD of PR8 NS1 (amino acid 1–73) and the effector domain (ED) (amino acid 74–230) and 5’ UTR derived from IA30 NS1 or vice versa were amplified from plasmids containing full-length NS genes of both strains [20] using a fusion PCR technique with a pair of specific primers. This strategy was designed to keep the NS2 transcripts intact. To make a single point mutation on NS1 protein of PR8, the NS1 sequences of PR8 and IA30 were compared to determine the different amino acids between the two strains. Four different amino acids on RBD were selected and a single point mutation was introduced into PR8 NS gene using specific primers. Amino acids at position 45, 55, 68 or 70 of PR8 NS1 were replaced with those of IA30. The mutations did not interfere with NS2 expression as they are spliced out to generate the mature mRNA. The dsRNA-binding deficient NS1, R38AK41A (AA) was constructed using similar strategies. The mutated NS genes were digested and inserted into the bidirectional vector pHW2000 [22].

The RBD (amino acids 1–73) of PR8 NS1 genes were cloned into the pQE80L vector (Qiagen) for protein expression in *E. coli* strain BL21. The DNA sequences in the positive clones were verified by DNA sequencing (Macrogen, Korea).

### Generation of viruses by reverse genetics

The viruses used in this study were generated by transfection using the eight-plasmid reverse genetics system in 293 T and MDCK cells as described previously [20, 42]. The plasmids used were as follows; NS1 from PR8 (WT or mutated PR8 NSs; G45R, K55E, I68V, K70E and AA), HA and NA from X31 or A/Hong Kong/1/1968 (H3N2) and the remaining five genes were from PR8. Briefly, 0.5 μg of each of the eight plasmids were incubated with 12 μl of X-tremeGENE9 transfection reagent (Roche) in 200 μl reaction for 15 min before they were gently overlaid onto a 50:50 293 T-MDCK cell mixture. The transfected cells were cultured in Opti-MEM I reduced serum (Gibco, Invitrogen) containing 1 μg/ml of TPCK-treated trypsin and incubated at 37 °C with 5 % CO_2_. The reverse genetics viruses were recovered by collecting supernatant at 48–72 hpi, which was then used to inoculate the allantoic cavities of 9-day-old specific pathogen free (SPF) embryonated chicken eggs. The existence of the mutation on each NS1 was confirmed by sequencing.

### Virus infection

The rescued viruses with different PR8 NS1 (WT, G45R or AA/NS1s) were used to infect A549 cells grown in 6-well plates in triplicate wells at an MOI of 0.01 or 2. The infected cells were maintained in Kaighn’s Modification of Ham’s F-12 Medium (ATCC) supplemented with 0.3 % BSA (Sigma-Aldrich), penicillin/streptomycin (Invitrogen) plus 1 μg/ml of TPCK-treated trypsin and incubated at 37 °C with 5 % CO_2_.

The viruses encoding WT, G45R and AA/NS1s were used to infect WT or IFNAR^null^ Let1 cells grown in 6-well plates in triplicate at an MOI of 1 or 1. The numbers of plaque forming units (pfu) in the inoculum were re-examined by plaque assay to confirm the equality of the virus in each inoculum (Additional file 4: Fig. S4). The infected cells were maintained in DMEM (Gibco, Invitrogen) supplemented with 0.3 % BSA (Sigma), penicillin/streptomycin (Gibco, Invitrogen) plus 0.1 μg/ml of TPCK trypsin (Sigma) and incubated at 37 °C with 5 % CO_2_. The cell supernatants were collected at indicated time points. The viruses collected at each time point were titrated by tissue culture infectious dose (TCID_50_) assay in MDCK cells and calculated as previously described [43].

### Expression of NS1 RBD

*E. coli* strain BL21 transformed with pQE plasmids containing the amino acid 1–73 of the RBD of PR8 NS1 including WT or mutants G45R and AA were grown in Luria broth with 100 mg/ml ampicillin (Invitrogen). Protein expression was induced with 0.2 mM IPTG (Invitrogen) at 37 °C for 18 h. The cells were then pelleted by centrifugation at 4000 rpm at 4 °C for 20 min. The cell pellets were mixed well in 30 μl of lysis buffer (1 M Tris, 300 mM NaCl, 1 % Triton X and 1 mM EDTA*,* pH 8.0). Subsequently, lysozyme and PMSF were added into the mixture at the final concentrations of 0.25 mg/ml and 1 mM, respectively. The mixture was vortex-mixed and incubated on ice for 30 min. The cells suspension was homogenized by sonication followed by centrifugation at 10,000 g at 4 °C for 20 min and the supernatant containing the soluble fraction of RBD NS1 was collected. The proteins were purified using Protino^®^ Ni-TED Resin-gravity-flow column (Macherey-Nagel) under native condition and were then concentrated in a 3 kDa-Centrifugal filter units (Millipore). The presentation of His-RBD NS1 proteins were examined by SDS-PAGE using 15 % acrylamide gels. Western blot analysis was performed using anti-NS1 rabbit hyperimmune serum and goat anti-rabbit IgG-HRP (Millipore) as primary and secondary antibodies, respectively.

### dsRNA-protein pull down assay

To generate dsRNA labeled with biotin, the biotinylated sense and antisense single-stranded (ss) RNA were in vitro transcribed. Briefly, the dsDNA template was produced by amplification of a 72 bp DNA fragment using forward and reverse primers containing the promoter sequences for the binding of T7 and T3 RNA polymerases, respectively [44]. The dsDNA fragment was purified using a DNA purification kit (Qiagen) and used as the template to produce biotinylated ssRNA by in vitro transcription using the Biotin RNA Labeling Mix (Roche) plus T7 or T3 RNA polymerase (Promega). The remaining DNA in the in vitro transcription reaction was destroyed by using DNase I (Promega) and the biotinylated ssRNA was purified by using ethanol precipitation method. The purified biotinylated T7- and T3-ssRNA were annealed at room temperature to obtain the biotinylated dsRNA and kept on ice until used in the binding reaction.

To optimize the pull down reaction, various concentrations of biotinylated dsRNA and NS1 RBD were examined in a checker board titration. Additional file 5: Fig. S5 shows an example of the titration results. The optimal condition was selected. In the pull down assay, 0.4 mg of each purified NS1 was incubated with 0.6 μM biotinylated dsRNA in 20 μl of binding buffer containing 50 mM Tris–HCl, pH 8.3, 75 mM KCl, 3 mM MgCl_2_, 8 % (v/v) glycerol, 0.1 % (v/v) Triton^®^ X-100 (Amresco), 1 mM DTT (Invitrogen), 5 μg yeast tRNA (Roche) and 40 Unit RNase OUT (Invitrogen). The reaction mixtures were incubated at room temperature without agitation for 30 min. After incubation, the dsRNA-NS1 complex was pulled down with NeutraAvidinAgarose Resin (Pierce, Thermo Scientific) by incubation at room temperature for 45 min. The resin was boiled in Lamelli sample buffer (Bio-Rad). The pulled-down protein was separated by using SDS-PAGE in 15 % gel. Western blot was performed by using anti-NS1 rabbit serum and goat-anti rabbit IgG-HRP (Millipore) as primary and secondary antibodies, respectively. The membrane was incubated in Clarity^®^ western ECL substrate (Bio-Rad) for 5 min. The chemiluminescent signal was detected by Fusion^®^ FX imaging analyzer (VilberLourmat).

### RIG-I mediated IFNβ-promoter activity assay

The plasmids expressing IFNβ-promoter (−125) firefly luciferase, *Renilla* Luciferase vector (pRL-TK; Promega), RIG-I and each PR8 NS1 (WT, G45R and AA) were co-transfected into 293 T cells grown in 24-well plate as previously described [45] with some modifications. Briefly, the indicated plasmids were transfected to the cells using XtremeGene HP transfection reagent (Roche). At 24 h post transfection (pt), the cells were lysed in passive lysis buffer (Invitrogen) and subjected for luciferase activity measurement using dual-luciferase kit following the manufacturer’s instruction (Promega). The firefly luciferase activity signals were normalized to the *Renilla* luciferase activity. The experiments were done in triplicate. NS1 expression levels in transfected cells were shown by western blot analysis (Additional file 2: Fig. S2).

### Quantitative real-time PCR (qRT-PCR)

Total RNA was isolated from A549 cells infected with the rX31 viruses encoding PR8 NS1 (WT and G45R) using Trizol^®^ reagent (Invitrogen) following the manufacturer’s instruction. Total RNA was isolated by using Direct-zol™ RNA MiniPrep (Zymo research). One microgram of total RNA was reverse transcribed using a high-capacity cDNA reverse transcription kit (Applied Biosystem). Realtime PCR was performed by using a 7500 Fast real-time PCR system (Applied Biosystem). In 15 μl reaction, 0.4 mM of forward and reverse primers and equal amount of cDNA were mixed with the SYBR^®^ Select master mix (Applied Biosystem) according to the manufacturer’s protocol. The qRT- PCR cycle were conducted under the following conditions: enzyme activation at 95 °C for 2 min followed by 40 cycles of denaturation at 95 °C for 3 s, annealing and extension at 60 °C for 30 s. Three biological replicates and two technical repeats were performed for each sample. The housekeeping gene, β-actin, was used as a reference control. The data of each sample and negative control (mock infected cells) were normalized to the reference using the threshold cycle (2^-*∆∆CT*^) method [46].

### Data analysis

Mean titers of the viruses at each time point, luciferase activity and relative mRNA expression level were compared and statistically calculated using analysis of variance (ANOVA) and student *t*-test method. Mean titers of the viruses as TCID_50_/ml was converted into base-10 logarithms and plotted against time points and a bar corresponding to SEM was demonstrated. A difference was considered significant if the *p*-value was < 0.05.

## Additional files

Additional file 1: Fig. S1. Three replicates of dsRNA-RBD pulled down assay. 0.6 μM of biotinylated-dsRNA and 0.4 mg NS1-RBDs were used in the pulled down assay. The assay was performed using similar condition in three separated reactions. The pulled-down NS1-RBD protein was electrophoresed through 15 % acrylamide gel and blotted onto a nitrocellulose membrane. Western blot was performed by using anti-NS1 rabbit serum and goat-anti rabbit IgG-HRP as primary and secondary antibodies, respectively. The protein was detected by chemiluminescent method. The G45R bound to dsRNA with lower capacity than the wild type (WT). The R38AK41A (AA) served as a negative control failed to bind with dsRNA. (TIFF 305 kb) Additional file 2: Fig. S2. NS1 expression in transfected 293 T cells as examined by western blot analysis. At 24 h post transfection, the cells were lysed in passive lysis buffer (Invitrogen) and subjected for western blot analysis to measure NS1 levels. The lysates were mixed in Laemmli sample buffer (Bio-Rad) and denatured at 95 °C for 5 mins prior to perform SDS-PAGE gel electrophoresis. Immunoblots were probed for NS1 protein (polyclonal rabbit anti-NS1; Thermoscientific) and β-actin (rabbit anti-actin antibody; Thermoscientific). Antibodies were detected by incubation with goat anti-rabbit HRP-linked antibody (Millipore). Lane 1 = empty plasmid; Lanes 2 and 3 = WT and G45R at 0.4 μg; Lanes 4 and 5 = WT and G45R at 0.2 μg. (TIF 105 kb) Additional file 3: Fig. S3. Western blot analysis of STAT1, pSTAT1 and NS1 protein. A549 cells were infected with rX31 encoding WT and G45R/NS1 viruses at an MOI of 2. At 8 h post infection, cell lysates were clarified by centrifugation at 13,000 rpm for 15 min. The lysates were mixed in Laemmli sample buffer (Bio-Rad) and denatured at 95 °C for 5 mins prior to perform SDS-PAGE gel electrophoresis. Immunoblots were probed for STAT1 (purified rabbit anti-Stat1 N-terminus, BD Transduction Laboratories), pSTAT1 (purified mouse anti-Stat1 pY701 BD Transduction Laboratories), NS1 (polyclonal rabbit anti-NS1; Thermoscientific) and actin (purified mouse anti-actin Ab-5; BD Transduction Laboratories). Antibodies were detected by incubation with goat anti-rabbit (GE healthcare) or goat anti-mouse HRP-linked antibody (Cell signaling). The immunoblots were visualized by using ChemiDoc XRS imager (Bio-Rad). Band intensity of proteins was quantified by using Image Lab version 5.0 (Bio-Rad). (TIFF 2319 kb) Additional file 4: Fig. S4. Plaque assay of the virus inoculums. The virus inoculums (WT and G45R/NS1) at MOI of 0.01 were serially diluted before they were infected onto MDCK cells to confirm that each inoculum had equal amount of the virus. Plaque assay of the viruses at 10^−5^ dilution is shown. The average virus titers of both WT and G45R/NS1 inoculums are 5× 10^6^ pfu/ml. (TIFF 1781 kb) Additional file 5: Fig. S5. Checker board titration of dsRNA and NS1-RBD. Different concentrations of biotinylated-dsRNA and NS1-RBD were titrated in the dsRNA-protein pulled down assay. The pulled-down NS1-RBD protein was separated by SDS-PAGE in 15 % acrylamide gel and blotted onto a nitrocellulose membrane. Western blot was performed by using anti-NS1 rabbit serum and goat-anti rabbit IgG-HRP as primary and secondary antibodies, respectively. The protein was detected by chemiluminescent method. Lanes 1, 2 and 3 are the reactions containing 0.4 μM dsRNA and 0.2, 0.4 and 0.6 mg NS1-RBDs. Lanes 4, 5 and 6 are the reactions containing 0.6 μM dsRNA and 0.2, 0.4 and 0.6 mg NS1-RBDs, respectively. N1 and N2 are the negative control reactions without dsRNA or RBD, respectively. (TIFF 204 kb)
