# Supplementary figures and images for: G45R mutation in the nonstructural protein 1 of A/Puerto Rico/8/1934 (H1N1) enhances viral replication independent of dsRNA-binding activity and type I interferon biology
Source: Virol J. 2016 Jul 12;13:127. doi: 10.1186/s12985-016-0585-4 (PMC4942902; doi:10.1186/s12985-016-0585-4)

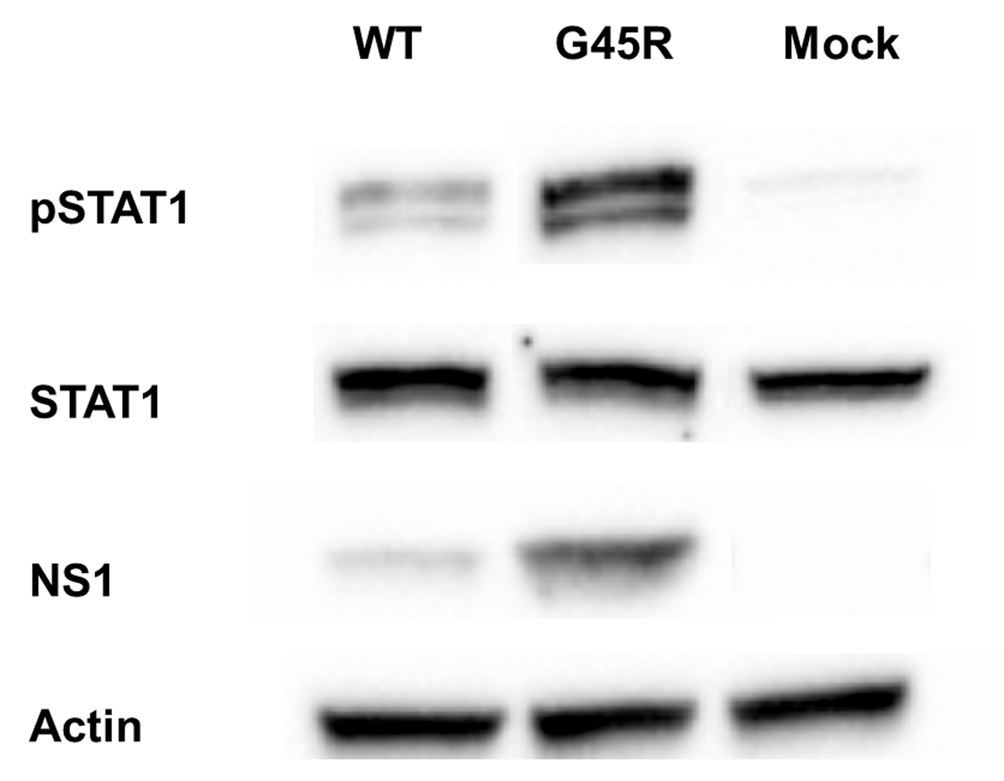

Supplement: Additional file 3: Fig. S3. — Western blot analysis of STAT1, pSTAT1 and NS1 protein. A549 cells were infected with rX31 encoding WT and G45R/NS1 viruses at an MOI of 2. At 8 h post infection, cell lysates were clarified by centrifugation at 13,000 rpm for 15 min. The lysates were mixed in Laemmli sample buffer (Bio-Rad) and denatured at 95 °C for 5 mins prior to perform SDS-PAGE gel electrophoresis. Immunoblots were probed for STAT1 (purified rabbit anti-Stat1 N-terminus, BD Transduction Laboratories), pSTAT1 (purified mouse anti-Stat1 pY701 BD Transduction Laboratories), NS1 (polyclonal rabbit anti-NS1; Thermoscientific) and actin (purified mouse anti-actin Ab-5; BD Transduction Laboratories). Antibodies were detected by incubation with goat anti-rabbit (GE healthcare) or goat anti-mouse HRP-linked antibody (Cell signaling). The immunoblots were visualized by using ChemiDoc XRS imager (Bio-Rad). Band intensity of proteins was quantified by using Image Lab version 5.0 (Bio-Rad). (TIFF 2319 kb) [file 12985_2016_585_MOESM3_ESM.tiff]

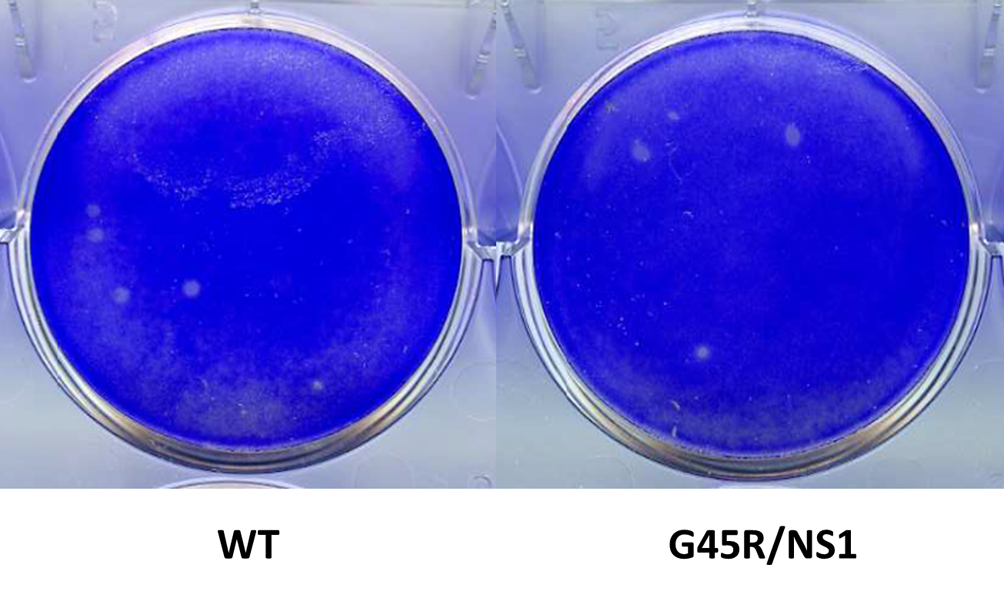

Supplement: Additional file 4: Fig. S4. — Plaque assay of the virus inoculums. The virus inoculums (WT and G45R/NS1) at MOI of 0.01 were serially diluted before they were infected onto MDCK cells to confirm that each inoculum had equal amount of the virus. Plaque assay of the viruses at 10−5 dilution is shown. The average virus titers of both WT and G45R/NS1 inoculums are 5× 10^6^ pfu/ml. (TIFF 1781 kb) [file 12985_2016_585_MOESM4_ESM.tiff]
